# Radioresistant Nasopharyngeal Carcinoma Cells Exhibited Decreased Cisplatin Sensitivity by Inducing SLC1A6 Expression

**DOI:** 10.3389/fphar.2021.629264

**Published:** 2021-04-13

**Authors:** Wenwen Hao, Lisha Wu, Linhui Cao, Jinxiu Yu, Li Ning, Jingshu Wang, Xiaoping Lin, Yanfeng Chen

**Affiliations:** ^1^ Department of Nasopharyngeal Carcinoma, State Key Laboratory of Oncology in South China, Collaborative Innovation Center for Cancer Medicine, Guangdong Key Laboratory of Nasopharyngeal Carcinoma Diagnosis and Therapy, Sun Yat-sen University Cancer Center, Guangzhou, China; ^2^ Department of Head and Neck Surgery, State Key Laboratory of Oncology in South China, Collaborative Innovation Center for Cancer Medicine, Sun Yat-sen University Cancer Center, Guangzhou, China; ^3^ Department of Oncology, Sun Yat-sen Memorial Hospital, Sun Yat-sen University, Guangzhou, China; ^4^ Department of Traditional Chinese Medicine, Sun Yat-sen Memorial Hospital, Sun Yat-sen University, Guangzhou, China; ^5^ Department of Radiotherapy, The Second Affiliated Hospital of Guangzhou Medical University, Guangzhou, China; ^6^ Department of Nuclear Medicine, State Key Laboratory of Oncology in South China, Collaborative Innovation Center for Cancer Medicine, Sun Yat-sen University Cancer Center, Guangzhou, China

**Keywords:** nasopharyngeal carcinoma, SLC1A6, cisplatin, radiation-resistance, glutamate

## Abstract

Cisplatin-based regimens are commonly used for the treatment of nasopharyngeal carcinoma (NPC) in patients who receive concurrent chemoradiotherapy. The sensitivity of NPC cells to cisplatin is closely associated with the efficacy of radiation therapy. In this study, we established two radioresistant NPC cell lines, HONE1-IR and CNE2-IR, and found that both cell lines showed reduced sensitivity to cisplatin. RNA-sequence analysis showed that SLC1A6 was upregulated in both HONE1-IR and CNE2-IR cell lines. Downregulation of SLC1A6 enhanced cisplatin sensitivity in these two radioresistant NPC cell lines. It was also found that the expression of SLC1A6 was induced during radiation treatment and correlated with poor prognosis of NPC patients. Notably, we observed that upregulation of SLC1A6 led to elevating level of glutamate and the expression of drug-resistant genes, resulted in reduced cisplatin sensitivity. Our findings provide a rationale for developing a novel therapeutic target for NPC patients with cisplatin resistance.

## Introduction

Nasopharyngeal carcinoma (NPC) is a malignant tumor in the head and neck with high incidence in southern China and Southeast Asia (Chen et al., 2019; Ji et al., 2019). According to the guidelines of the National Comprehensive Cancer Network (NCCN), radiotherapy and cisplatin-based regimens are the main treatments for NPC patients (Pfister et al., 2020). The cisplatin-based concurrent chemoradiotherapy has been proven to improve the outcome of early and locally advanced NPC (Chen et al., 2011). However, there are a small portion of patients who did not response effectively or became recurrent to these treatments, and their prognosis remains poor (Chen et al., 2011; Karam et al., 2016).

The antitumor mechanism of cisplatin is to form covalent DNA adducts thus interfering with DNA repair (Wang and Lippard, 2005). The combination of cisplatin provides a synergetic effect for radiotherapy for the reason that cisplatin could enhance the sensitivity to radiation (Boeckman et al., 2005). Interestingly, some studies have revealed that cancer cells could acquire cisplatin resistance after radiation therapy (Eichholtz-Wirth, 1995; Zhuang et al., 2019). Based on our clinical observations, the phenomenon of cisplatin resistance is commonly seen in NPC patients who also resistant to radiotherapy. However, the association of cisplatin and radiation resistance is elusive.

The SLC1A6 (Solute Carrier Family 1 Member 6) is a member of the SLC1A family, which consists of the excitatory amino acid transporters EAAT1–EAAT5 (designated as SLC1A1-3, 6-7) and the alanine serine cysteine transporters ASCT1-ASCT2 (designated as SLC1A4-5) in mammals (Freidman et al., 2020). This transmembrane transporter encoded by SLC1A6 mediates the uptake of l-glutamate and L/d-aspartate (Fairman et al., 1995; Ryan et al., 2009; Vandenberg and Ryan, 2013; Guskov et al., 2016). Although the role of SLC1A6 in cancers was not well documented, other members of the SLC1A family were reported to be overexpressed in multiple tumors and predict poor prognosis. For example, expression of SLC1A1, SLC1A2 or SLC1A3 contributed to promoting tumor progression in solid tumors, such as lung cancer, glioma, and gastric cancer (Ye et al., 1999; de Groot et al., 2005; Tao et al., 2011; Xu et al., 2020; Guo et al., 2021). Besides, the SLC1A members were also reported to be associated with drug resistance. SLC1A1 was upregulated in oxaliplatin-resistant colorectal cancers (Pedraz-Cuesta et al., 2015), and SLC1A3 was associated with l-asparaginase resistance in acute lymphoblastic leukemia (Sun et al., 2019). As mammalian transporters of amino acids, members of the SLC1A family are implicated to impact drug-related metabolic profiles in tumor cells.

In the present study, we established two radioresistant NPC cell lines, HONE1-IR and CNE2-IR. We found that the radioresistant NPC cells acquired the characteristic of reduced cisplatin sensitivity, which was associated with the upregulation of SLC1A6. By inducing SLC1A6, HONE1-IR and CNE2-IR cells increased the cellular glutamate level and drug resistance genes, leading to reduced cisplatin sensitivity.

## Materials and Methods

### Ethical Statement

The approval of this study was obtained from the Ethics Committee of Sun Yat-sen University Cancer Center (SYSUCC, Guangzhou, China). This study met the ethical standard of the Declaration of Helsinki. The nasopharyngeal biopsy was performed in all patients who have submitted their informed consent.

### Patients and Specimens

A total of 78 NPC patients treated in Sun Yat-sen University Cancer Center were recruited and completely followed up from February 2011 to August 2014. The NPC biopsy specimens were fixed in 4% paraformaldehyde and paraffin-embedded and used for immunohistochemical (IHC) analysis of SLC1A6 expression. SLC1A6 expression was scored according to the staining intensity and percentage of positively stained cells. The staining intensity score of SLC1A6 was graded as follows: 0, no staining; 1, weak staining (light yellow); 2, moderate staining (yellow-brown); 3, intense staining (brown). The staining percentage score of SLC1A6 was graded as follows: 1, percentage of positive cells less than 30%; 2, percentage of positive cells between 30 and 60%; 3, percentage of positive cells more than 60%. The total-score was calculated as the formula: Total score = ∑ (Intensity score × percentage score)=(1 × percentage score) + (2 × percentage score) + (3 × percentage score). Finally, the intensity score, percentage score, and total score were used to verify the prognostic value of SLC1A6 expression for overall survival (OS).

### Immunohistochemistry

The 5-μm paraffin sections were deparaffinized with xylene and rehydrated in graded ethanol. Antigen retrieval was achieved by placing the sections in sodium citrate buffer (pH 6.0) at 95°C for 20 min. The sections were blocked with 5% goat serum in PBS and incubated with primary antibody against SLC1A6 (1:100, Thermo fisher, the United States) overnight at 4°C. The next day, the sections were stained with SP-9000 Detection Kits (Biotin-Streptavidin HRP Detection Systems, ZSGB-Bio, China) and the DAB Kit (ZSGB-Bio, China) was used for color development according to the manufacturer’s manual. The sections were counterstained with hematoxylin and observed with a light microscope.

### Cells and Cell Culture

Human NPC cell lines CNE2 and HONE1 were gifted from Professor Chaonan Qian (SYSUCC) (Guo et al., 2020). Both the CNE2 and HONE1 cell lines, and their radioresistant cell lines HONE1-IR and CNE2-IR, were cultured in RPMI-1640 medium (Gibco, Thermo fisher, United States), supplemented with 10% fetal bovine serum (FBS, Gibco) and 1% antibiotics (Penicillin-Streptomycin). Another head and neck cell line SCC9 was cultured in F12-Dulbecco’s modified Eagle’s medium (Gibco, Thermo fisher, United States), supplemented with 10% FBS and 1% antibiotics (Penicillin-Streptomycin). Customized RPMI-1640 (deprived glutamic acid or aspartic acid) was ordered from Weiga Biotechnology Company (Guangzhou, China), supplemented with 10% dialyzed FBS (Gibco, Thermo fisher, United States). All cells were incubated in a humidified atmosphere with 5% CO_2_ at 37°C.

### Establishment of Radioresistant Cell Lines

According to our previous study (Guo et al., 2020), HONE1 cells were cultured in RPMI-1640 medium supplemented with 10% FBS and reached approximately 50% confluence in 25-cm^2^ flasks. Cells were treated with a dose of 6Gy radiation using an X-rays generator. After radiation, the culture medium was replaced with complete fresh medium and cells were returned to the incubator. Cells were passaged until they reached approximately 90% confluence. The fractionated irradiations were repeated five times and reached a total dose of 30Gy. The interval between each radiation was at least 2-weeks for all cells. Radioresistant cell population were selected and were referred as HONE1-IR cells. The parental cells without irradiation were used as control cells. The CNE2-IR cells were kindly provided by Professor Yunfei Xia (SYSUCC).

### Quantitative Real-Time PCR

The total RNAs were extracted by the TRIzol reagents (RNAiso PLUS, Invitrogen, US) following the manufacturer's manuals. The isolated RNAs were assessed by Bioanalyzer 2,200 (Agilent, Uunited States) to determine their concentrations and quality before conversion to cDNA. Quantitative RT-PCR was carried out using the Real-Time PCR Detection System (Lightcycler 480 II, Roche, United States) based on the manufacturer's manual. Values were expressed as fold changes of the controls using the 2e^−ΔΔCt^ method.

### RNA-Seq Transcriptome Analysis

NPC Cells (HONE1 and HONE1-IR, CNE2, and CNE2-IR) were cultured and their total RNAs were extracted as described above and kept at −80°C. Sequencing and bioinformatic analysis were performed by DESeq2 and edgeR platforms with the aid of Novogene Company (Beijing, China). The intensity was used to generate the heatmap by Novomagic platform (Novogene Company). Differentially expressed genes were determined when *p*0.05 and the absolute log2 fold change of expression was greater than 3.

### MTS Assay

HONE1 (HONE1-IR and NC cells) and CNE2 (CNE2-IR and NC cells) were planted into 96-well plates at 1–2 × 10^3^ cells/200 μl/well, treated with increasing concentration of cisplatin for 24 h. In the last 2 h of incubation, 20 μL of MTS tetrazolium (Promega, United States) was added to each well based on the manufacturer's protocols. Cell viability was examined by assessing the light absorbance at 490–500 nm. The cell survival curves were drawn based on the results obtained.

### Colony Formation

HONE1 (HONE1-IR cells and NC cells) and CNE2 (CNE2-IR and NC cells) were planted into 6-well plates at 5 × 10^2^ cells/well. Subsequently, cisplatin was added to the cultured medium at the concentration of 20 μM for 24 h of treatment. After that, the culture medium was replaced by a fresh medium. The cells were cultured for 2 weeks. Crystal violet was used to stain the colonies.

### Small Interfering RNA Transfection

HONE1-IR and CNE2-IR cells were planted into 6-well plates one day before transfection to reach about 60–70% confluence. The siRNAs targeting SLC1A6 gene were purchased from Ruibo Company (Guangzhou, China). Transient transfection of HONE1-IR and CNE2-IR cells was performed using Lipofectamine RNAiMax (Invitrogen, United States) according to the manufacturer's protocols. Cells were transfected with a total of 50 pmol siRNA and subjected to Western blot and MTS assays after 24–48 h post-transfection. The siRNA sequences for SLC1A6 were as follows:

SLC1A6-siRNA-15′-AUGAAAACUGCAAUGACUGUA-3′

SLC1A6-siRNA-25′-AAGGAAUAAGCCAACGAUGAC-3′

### Establishment of SLC1A6 Overexpressed Stable Cell Lines

The cDNA of SLC1A6 was synthesized according to the human full-length open reading frame of SLC1A6 mRNA (NM_001,272,087) and integrated into pcDNA3.1 plasmid. The lentiviral expression plasmid was used to transfect 293T cells for packaging. The culture media of transfected 293T cells was harvested and used to transfect HONE1 or CNE2 cells. Cells expressing SLC1A6 were selected with puromycin (Sigma-Aldrich, United States) in the concentration of 2 µg/ml. SLC1A6-overexpressed stable cells were established after 10 days selection.

### Western Blotting Analysis

Following treatments, cells were lyzed by RIPA buffer (Beyotime, China) containing protease and phosphatase inhibitors (Life Technologies, United States). Protein concentrations were detected by a BCA kit (Thermo Fisher, United States). Proteins of samples were subjected to 8–10% SDS-PAGE and transferred to PVDF membranes (Biorad, United States). After being blocked with 5% non-fat milk for 1 h, membranes were incubated with primary antibodies at 4°C overnight. Primary antibodies against SLC1A6 (1:1,000, Thermo fisher), γH2AX (1:1,000, Cell Signaling), Beta-actin (1:1,000, Cell Signaling) were used. Then the membranes were washed with TBS containing 0.1% Tween-20, followed by incubation with HRP anti-rabbit (1:1,000, Cell Signaling) secondary antibody. The band intensity values were normalized to that of Beta-actin.

### Measurement of Glutamate or Aspartate Level

A total of 10 × 10^6^ cells were collected and lyzed by ultrasonication (low frequency, 3s, 20 times), glutamate or aspartate content was extracted by glutamate assay kit (Solarbio, China) or aspartate assay kit (Abnova, Taiwan). The level of glutamate or aspartate was detected by comparing the light absorbance value with the standard solution curve. The absorbance value was measured at 340 nm or 570 nm, respectively.

### Online and Public Database

The correlation of SLC1A6 expression and survival outcome in patients with head and neck squamous cell carcinoma (HNSCC) in the TCGA database was analyzed on the GEPIA website (http://gepia.cancer-pku.cn/).

### Statistical Analysis

The data were expressed as the mean ± SD (the standard deviation). Comparisons between two groups were analyzed by unpaired Student’s *t* test. Comparisons among groups were performed by one-way analysis of variance (ANOVA) followed by Tukey’s test. Significant *p* value was considered as0.05. In all cases, *p < 0.05, **p < 0.01, ***p < 0.001.

## Results

### Radioresistant HONE1-IR and CNE2-IR Cells Showed Reduced Sensitivity to Cisplatin

**FIGURE 1:**
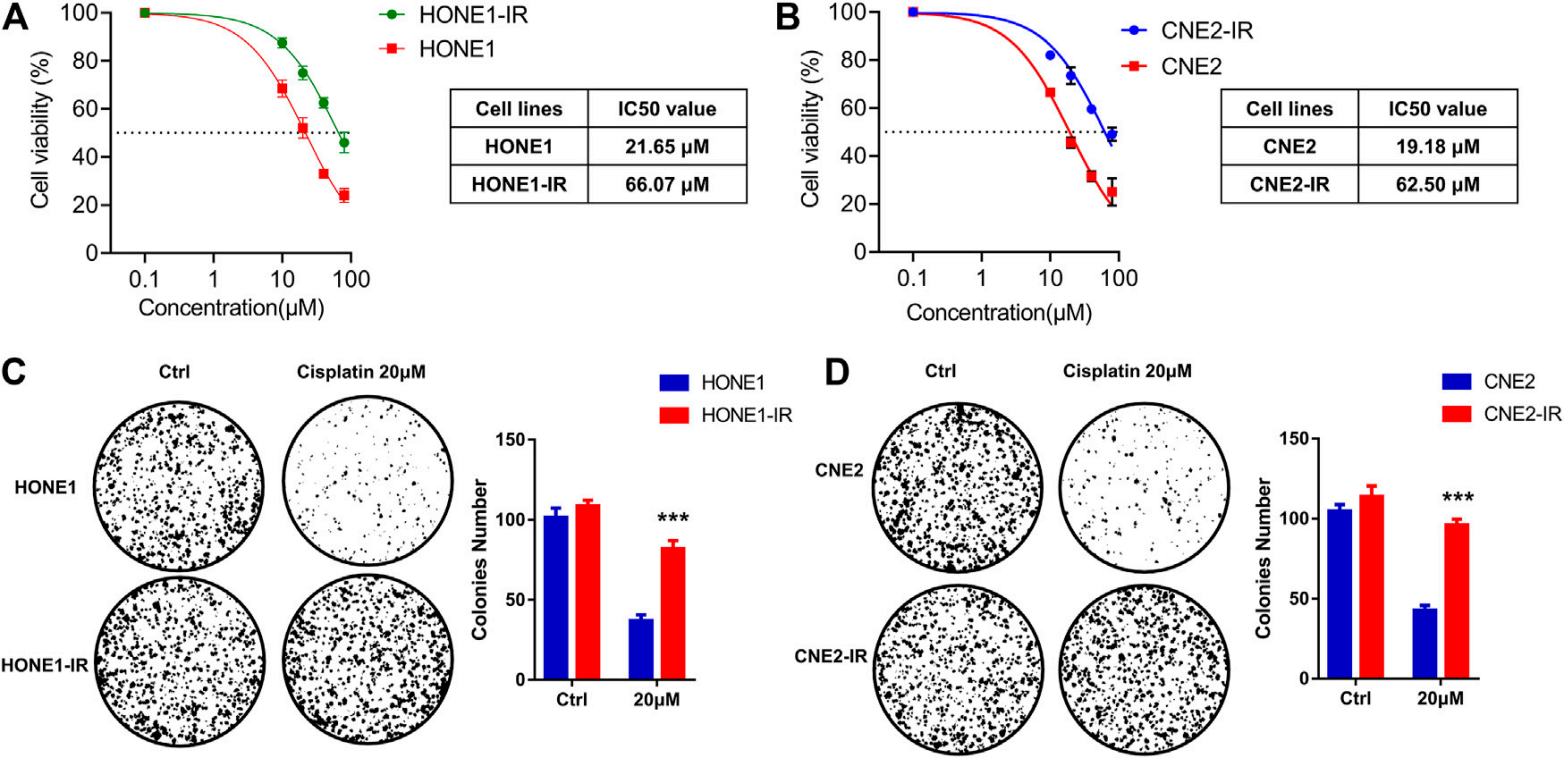
Radioresistant HONE1-IR and CNE2-IR cells showed reduced sensitivity to cisplatin. **(A–B)** MTS assay on the IC50 values of radioresistant NPC cell lines HONE1-IR and CNE2-IR, as well as their parental cell lines HONE1 and CNE2 with cisplatin treatment. ANOVA test **(C–D)** Colony formation assay on the radioresistant NPC cell lines and their parental cell lines. Unpaired Student’s t test. *p < 0.05, **p < 0.01, ***p < 0.001.

Two radioresistant human NPC cell lines, HONE1-IR and CNE2-IR, were generated and their resistance to radiation was verified by survival assay (Guo et al., 2020). As the sensitivity to cisplatin is closely associated with radiotherapy efficacy, we compared the sensitivity to cisplatin in radioresistant NPC and their parental cells. MTS assay supported that radioresistant NPC cells exhibited reduced sensitivity to cisplatin compared to their parental cells. Subsequent analysis of chemosensitivity indicated that the cisplatin IC50 of HONE1-IR cells was significantly higher than that of their parental cells (66.07 vs. 21.65 μM)). Similarly, CNE2-IR cells also showed higher IC50 than that of their parental cells (62.50 vs. 19.18 μM) (Figures 1A,B). Colony formation assay showed that cisplatin treatment significantly reduced tumor cell proliferation in HONE1 and CNE2 cells, but not in HONE1-IR and CNE2-IR cells (Figures 1C,D). These findings indicated that radioresistant NPC cells reduced cisplatin sensitivity.

### SLC1A6 Gene was Up-Regulated in Both HONE1-IR and CNE2-IR Cells

**FIGURE 2:**
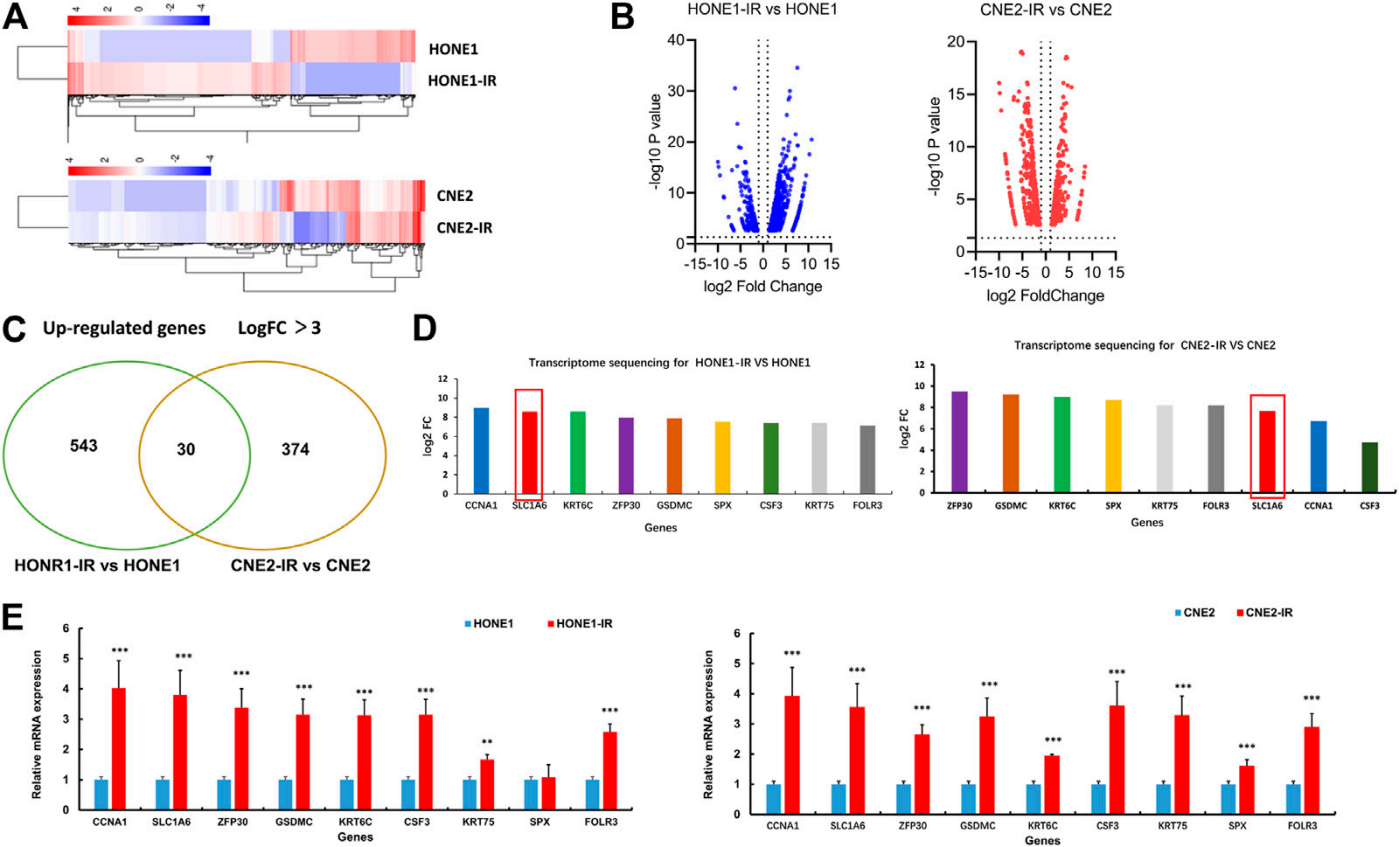
SLC1A6 up-regulated in radioresistant NPC cells. **(A)** Heat maps showing the expression pattern of up-regulated and down-regulated genes in HONE1 cells (HONE1-IR vs. HONE1) and CNE2 cells (CNE2-IR vs. CNE2). Red or blue represents high or low expression, respectively (Raw Z score). **(B)** Volcano maps showing the expression pattern of genes. **(C)** Venn maps showing 573 up-regulated genes in HONE1R vs. HONE1 cells and 404 up-regulated genes in CNE2-IR vs. CNE2 cells, with 30 common genes. **(D)** Transcriptome sequencing showing the highest fold change in up-regulated nine genes, co-expressed in both radioresistant NPC cells, including SLC1A6. **(E)** qRT-PCR verified the different mRNA expression of the nine genes. Unpaired Student’s t test. *p < 0.05, **p < 0.01, ***p < 0.001.

To explore the potential regulators of cisplatin sensitivity in radioresistant NPC cells, RNA-Sequence was performed to compare the transcriptome profile of HONE1-IR cells and their parental cells. The cutoff criteria were more than 3-fold or less than 3-fold for upregulation or downregulation, respectively. As for CHE2-IR cells and their parental cells, the same method was performed. Heatmap showed a total of 573 genes in HONE1 cells (HONE1-IR cells vs. their parental cells) and 404 genes in CNE2 cells (CNE2-IR cells vs. their parental cells) were identified to be differentially expressed (Figures 2A,B). There were 30 common genes upregulated in both HONE1-IR and CNE2-IR cells compared to their parental cells (Figure 2C). SLC1A6 gene was particularly noted in the top nine over-expressed genes (Figure 2D) and verified by qRT-PCR (Figure 2E). These results indicated that SLC1A6 might be a crucial gene associated with cisplatin or radiation sensitivity in radioresistant NPC cells.

### SLC1A6 Overexpression Conferred Reduced Cisplatin and Radiation Sensitivity in Radioresistant NPC Cells

**FIGURE 3:**
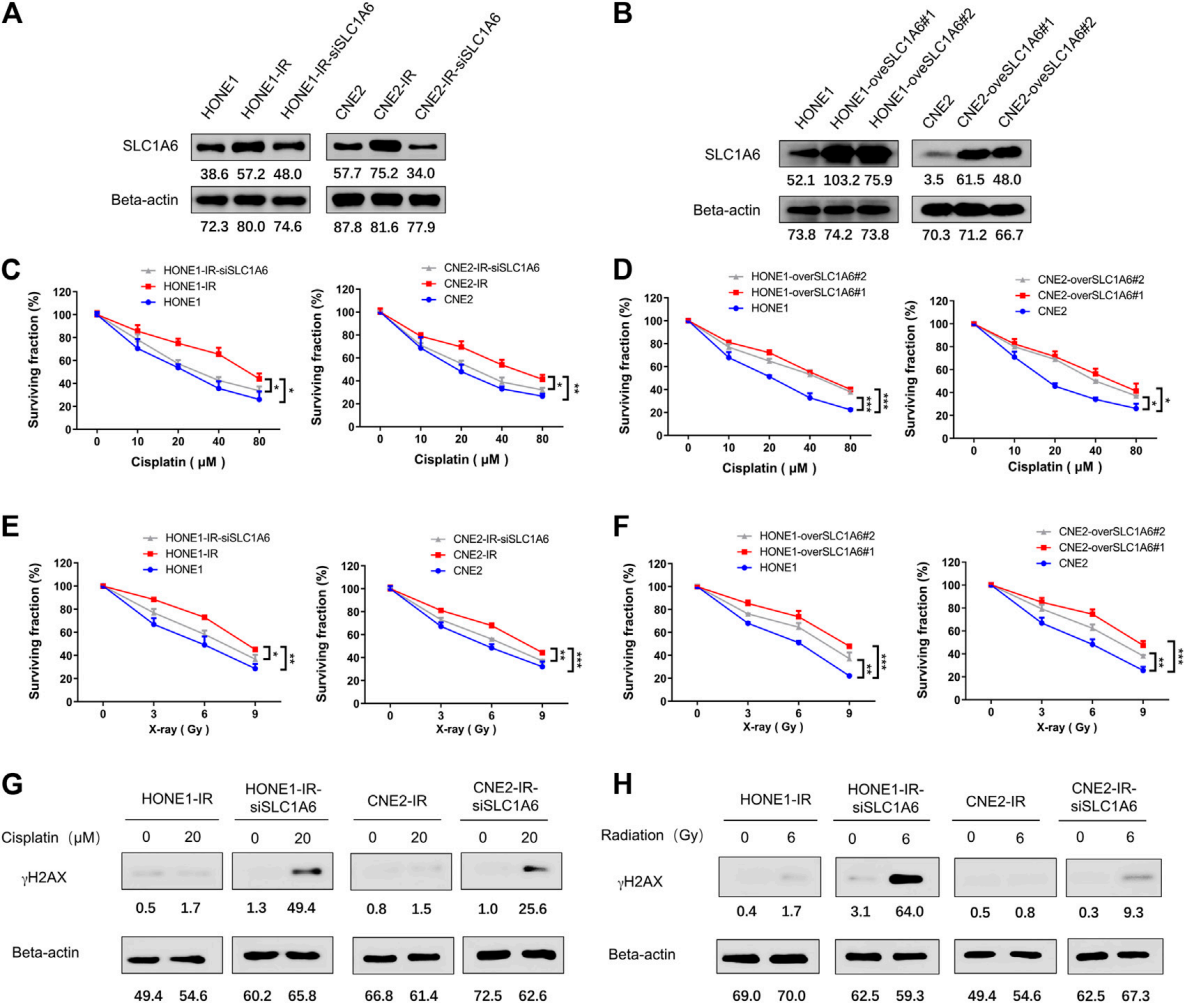
SLC1A6 mediated reduced sensitivity to cisplatin and radiation in radioresistant NPC cells. **(A)** SLC1A6 and Beta-actin protein expression of HONE1, CNE2, HONE1-IR, CNE2-IR and radioresistant NPC SLC1A6 knockdown cell lines, detected by Western blots. **(B)** SLC1A6 and Beta-actin protein expression of HONE1, CNE2, HONE1-overSLC1A6 and CNE2-overSLC1A6 cell lines, detected by Western blots. **(C–D)** MTS assays conducted in HONE1, CNE2, HONE1-IR, CNE2-IR, radioresistant NPC SLC1A6 knockdown cell lines or SLC1A6 overexpressed cell lines, treated with cisplatin. The difference was analyzed by repeated ANOVA. **(E–F)** MTS assays conducted in HONE1, CNE2, HONE1-IR, CNE2-IR, radioresistant NPC SLC1A6 knockdown cell lines or SLC1A6 overexpressed cell lines, treated with fractional radiation. The difference was analyzed by repeated ANOVA. **(G–H)** The levels of γH2AX and Beta-actin analyzed by Western blots. *p < 0.05, **p < 0.01, ***p < 0.001.

Furthermore, the up-regulation of SLC1A6 has been verified by Western blots in both HONE1-IR and CNE2-IR cells compared to their parental cells (Figure 3A). To further investigate the role of SLC1A6 in NPC radioresistant cells, siRNA or SLC1A6 overexpression lentivirus was used to knock down or up regulate the expression of SLC1A6 in cells (Figure 3B). Results showed that downregulation of SLC1A6 gene re-sensitized radioresistant NPC cells to cisplatin treatment (Figure 3C). Moreover, overexpression of SLC1A6 decreased the sensitivity to cisplatin in parental cells (Figure 3D). Besides, modulation of the SLC1A6 also impacted the sensitivity to radiation in radioresistant NPC and parental cells (Figures 3E,F). Cisplatin and radiation both cause DNA damage. Increased gamma-H2AX (γH2AX) expression, a biomarker of DNA damage, was noticed in radioresistant NPC cells by knocking down SLC1A6, followed by cisplatin or radiation treatment (Figures 3G,H). These results demonstrated that SLC1A6 contributed to reducing cisplatin and radiation sensitivity of radioresistant NPC cells.

### SLC1A6 Induced by Radiation Treatment and Correlated with Poor Prognosis

**FIGURE 4:**
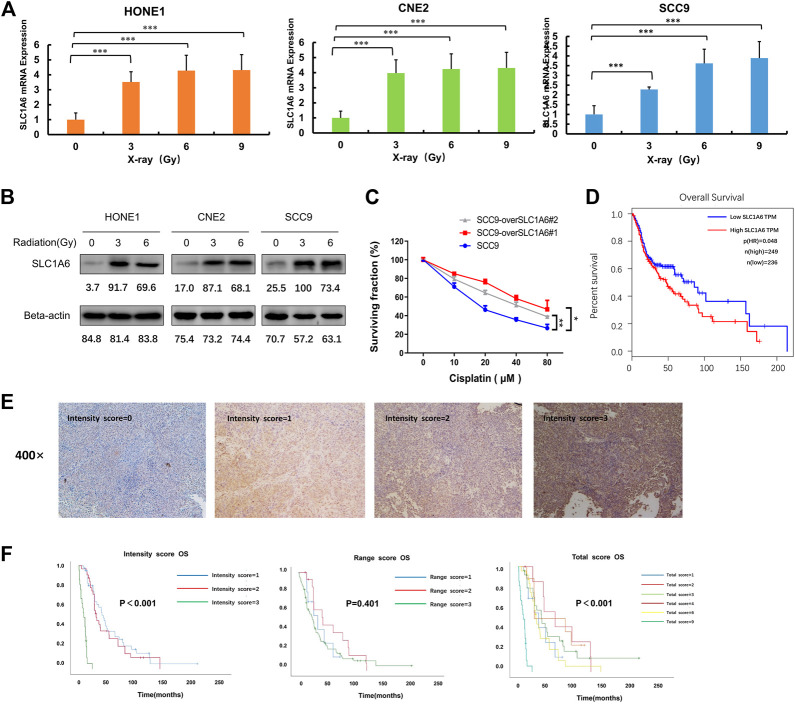
SLC1A6 induced by radiation treatment and correlated with poor prognosis. **(A)** The mRNA expression of SLC1A6 in HONE1, CNE2, and SCC9 cells treated with increasing radiation doses. **(B)** The protein expression of SLC1A6 in HONE1, CNE2, and SCC9 cells treated with increasing doses of radiation. **(C)** Cell viability examined by MTS assays in SCC9 cells treated with cisplatin. **(D)** Patients with SLC1A6 overexpression showed poor overall survival analyzed with GEPIA websites. **(E)** Representative micrographs (400x). All images were acquired and processed at identical conditions. **(F)** OS curves on the SLC1A6 expression in validation set consisting of 78 patients with NPC from Sun Yat-sen University Cancer Center, using intensity score, percentage score, or total score, respectively. The differences were measured by Kaplan–Meier log-rank test. *p < 0.05, **p < 0.01, ***p < 0.001.

As SLC1A6 gene was up-regulated in the radioresistant NPC cells, we next investigated the impact of radiation on SLC1A6. We observed both RNA (Figure 4A) and protein levels (Figure 4B) of SLC1A6 increased during radiation treatment in NPC cells. As NPC belongs to HNSCC, another HNSCC cell line SCC9, was utilized to further verify the role of the SLC1A6 gene on the sensitivity to cisplatin. Similarly, SLC1A6 up-regulation was seen during radiation treatment in SCC9 cells (Figures 4A,B). SLC1A6 over-expressed SCC9 cells exhibited low sensitivity to cisplatin (Figure 4C). Analysis on TCGA database showed that high SLC1A6 expression was correlated with poor prognosis in HNSCC patients (Figure 4D). To further validate the prognostic role of the SLC1A6 in NPC patients, we collected 78 biopsies from NPC patients in Sun Yat-sen University Cancer Center. We demonstrated that high SLC1A6 expression was correlated with poor prognosis in these patients (Figures 4E,F). These results demonstrated that overexpression of SLC1A6 was associated with low therapeutic efficacy and poor survival in NPC patients.

### SLC1A6 Up-Regulated Glutamate Level and Drug Resistance Genes

**FIGURE 5:**
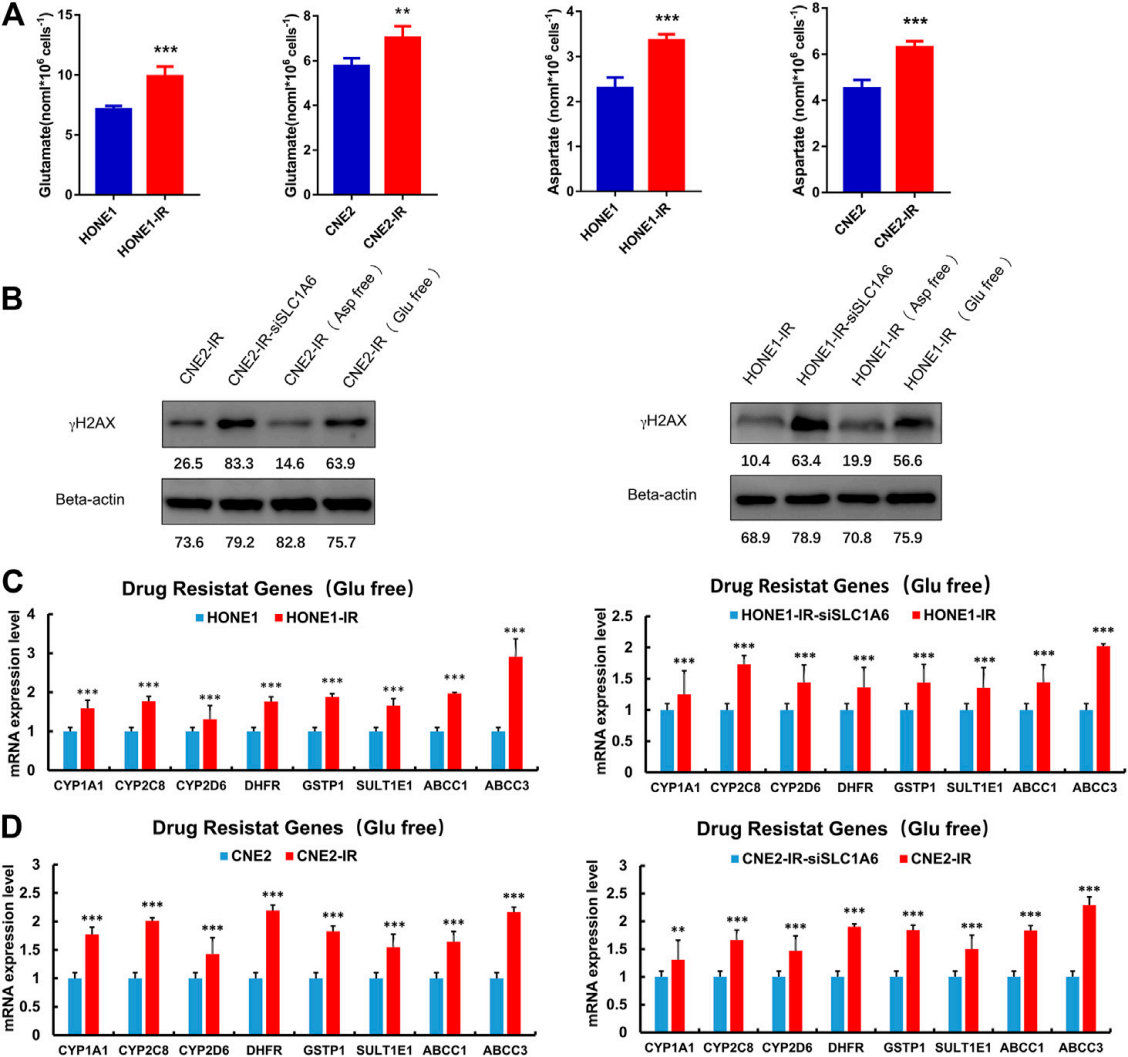
SLC1A6 increased glutamate level and drug resistance gene expression. **(A)** The level of glutamate and aspartate in radioresistant NPC cells and their parental cells. **(B)** The level of γH2AX and Beta-actin in radioresistant NPC cells, radioresistant NPC SLC1A6 knockdown cells, radioresistant NPC cells cultured with glutamic acid free medium and radioresistant NPC cells cultured with aspartic acid free medium, treated with cisplatin. **(C–D)** RT-PCR on the mRNA expression of drug resistance genes in NPC cells, radioresistant NPC cells and radioresistant SLC1A6 knockdown cells. Unpaired Student’s t test. *p < 0.05, **p < 0.01, ***p < 0.001.

Further experiments were conducted to elucidate the underlying mechanism of SLC1A6 in regulating cisplatin sensitivity in radioresistant NPC cells. SLC1A6 is one of the members of the EAATs family, which transport aspartate, glutamate, and cysteine. These amino acids serve as substrates in several biochemical and metabolic pathways in cancer cells. Previous studies have reported that the EAATs-mediated therapeutic resistance is related to altered tumor metabolic profiles (Ye et al., 1999; de Groot et al., 2005; Tao et al., 2011; Pedraz-Cuesta et al., 2015; Sun et al., 2019; Xu et al., 2020; Guo et al., 2021). It was found that the level of glutamate and aspartate increased in the radioresistant NPC cells compared to their parental cells (Figure 5A). In addition, deprivation of glutamate, not aspartate, in the culture medium re-sensitized radioresistant NPC cells to cisplatin treatment (Figure 5B). Moreover, PCR analysis revealed that the expression of CYP1A1, CYP2C8, CYP2D6, DHFR, GSTP1, and SULT1E1 genes, that are associated with drug catabolism, were significantly higher in radioresistant NPC cells compared to their parental cells (Figure 5C). ABCC1 and ABCC3, that are associated with drug transportation, were also elevated in radioresistant NPC cells (Figure 5C). The expressions of these genes were significantly decreased when SLC1A6 was knocked down in radioresistant NPC cells (Figure 5D). These results collectively supported that SLC1A6 overexpression reduced cisplatin sensitivity in radioresistant NPC cells by increasing the level of glutamate and drug resistance genes.

## Discussion

Radiation resistance has been a significant obstacle for the local control of NPC. Some patients who are resistant to radiotherapy are also not sensitive to cisplatin treatment. In this study, we observed the decreased sensitivity to cisplatin in radiation resistant NPC cells. Although previous studies have revealed the mechanism of radiation-resistance or cisplatin resistance, the relationship between radiation and cisplatin resistance is complicated and not elucidated. The cause of cisplatin resistance, included aberrant repair of DNA damage, apoptosis pathway defects, activation of drug export system, altered cellular metabolism, reduced oxidative stress, and cancer stem cell induction, etc. (Ikuta et al., 2005; Galluzzi et al., 2012; Zhang et al., 2012; Cruz-Bermúdez et al., 2019) As both radiation and cisplatin cause DNA damage in tumor cells, they might share the same biological pathway to reverse DNA damage. Cross-resistance mechanisms reported by studies include elevated GSH level, DNA repair enzymes, NFκB and TNFα, etc. (Chao et al., 1991; Poppenborg et al., 1997; Zhu et al., 2019)

Here, the identification of SLC1A6 as the crucial gene conferring reduced cisplatin sensitivity in radiation-resistant NPC cells is novel. In this study, it was found that SLC1A6 expression was upregulated in HONE1-IR and CNE2-IR cells. Down-regulating SLC1A6 expression could significantly rescue the cisplatin sensitivity in HONE1-IR and CNE2-IR cells. The SLC1A6 could be the common factor to reduce DNA damage from radiation or cisplatin treatment, which was confirmed by our study. Our results were in consistent with previous studies that described the radiation-induced cisplatin resistance (Eichholtz-Wirth et al., 1993; Eichholtz-Wirth, 1995; Zhuang et al., 2019). The cisplatin resistance acquired during radiation could explain the phenomenon that some patients with radio-resistance also didn’t respond to cisplatin treatment and correlated with the radiation-induced SLC1A6 upregulation. However, the mechanism of radiation induced SLC1A6 overexpression remained far from understood. We hypothesized that genetic reprogramming happened in the process of DNA injury and repair upon radiation.

SLC1A family is thought to contribute to tumor progression by regulating microenvironments and metabolic profiles (Ye et al., 1999; de Groot et al., 2005; Tao et al., 2011; Pedraz-Cuesta et al., 2015; Sun et al., 2019; Freidman et al., 2020; Xu et al., 2020; Guo et al., 2021). SLC1A6 transports aspartate, glutamate, and cysteine, and regulation of these amino acids is essential for numerous biochemical and metabolic pathways such as the TCA cycle or nucleotide synthesis. For example, glutamate could be transformed into glutamine, facilitating nucleotide synthesis and repair DNA damage (Fu et al., 2019). The endocrine resistance breast cancer cells would increase aspartate and glutamate import to sustain DNA, lipid, and protein synthesis (Bacci et al., 2019). We found both glutamate and aspartate were elevated in radioresistant NPC cells. However, only glutamate played a vital role in resisting DNA injury from cisplatin treatment. These findings suggested that SLC1A6 contributed to metabolic reprogramming in radioresistant NPC cells.

We also uncovered that SLC1A6 promoted the upregulation of drug catabolic genes (CYP1A1, CYP2C8, CYP2D6, DHFR, GSTP1, and SULT1E1) and drug transport genes (ABCC1 and ABCC3). The CYP enzymes have been extensively investigated in drug metabolism, and their inhibitors were proven to be effective in reversing cisplatin sensitivity in cancer cells (Sonawane et al., 2019). Gstp1, a GST family member, is involved in the detoxification of cisplatin via cisplatin-glutathione adducts formation (Li et al., 2019). DHFR and SULT1E1 have been reported to be up-regulated in cisplatin-resistant cells (Marverti et al., 2009; Varamo et al., 2019). The ABC family of transporters is referred to as multidrug resistance proteins that transport substrates across the cellular membranes (Chen et al., 2016). These results implicated that SLC1A6 contributed to cisplatin resistance in radioresistant NPC cells through multiple factors.

In summary, we identified the upregulation of SLC1A6 in radioresistant NPC cells. Overexpression of SLC1A6 is correlated with reduced sensitivity to cisplatin by elevating the level of glutamate and drug resistance genes. Targeting SLC1A6 could be a potential strategy to enhance cisplatin sensitivity in NPC patients.

## Data Availability

The datasets presented in this study can be found in online repositories. The names of the repository/repositories and accession number(s) can be found below: NCBI SRA, PRJNA700383.
